# FEDERAL ACTIONS TO SUPPORT FAMILY CAREGIVERS UNDER THE BIDEN ADMINISTRATION

**DOI:** 10.1093/geroni/igac059.138

**Published:** 2022-12-20

**Authors:** Eileen Tell

**Affiliations:** ET Consulting, LLC, Belmont, Massachusetts, United States

## Abstract

Family caregivers are the glue holding together the delivery and financing of long-term care. Replacing family care with paid care would cost roughly $470 billion each year. But family caregivers are struggling. They face many challenges – most notably the financial stress and the need for services and supports. Other challenges include lack of respite care, need for caregiver training, and lack of access to quality paid workforce. In order to address these challenges, Congress authorized the RAISE Family Caregiving Advisory Council. The RAISE (Recognize, Assist, Include, Support and Engage) Family Caregivers Act directs the Secretary of Health and Human Services to develop a national family caregiver strategy. This session presents the findings of two years of focus groups and interviews with family caregivers and hundreds of stakeholder organizations that support them, providing concrete input to the Biden administration on how to deliver on the broad objectives of the RAISE Act.

